# Hydroxyapatite from Fish for Bone Tissue Engineering: A Promising Approach

**DOI:** 10.22088/IJMCM.BUMS.7.2.80

**Published:** 2018-06-28

**Authors:** Renata Neves Granito, Ana Claudia Muniz Renno, Hirochi Yamamura, Matheus Cruz de Almeida, Pedro Luiz Menin Ruiz, Daniel Araki Ribeiro

**Affiliations:** 1 *Department of Biosciences, Federal University of São Paulo, UNIFESP, Santos, SP, Brazil.*; 2 *Department of Chemistry, Catholic University of Santos, UNISANTOS, Santos, SP, Brazil.*

**Keywords:** Fish, hydroxyapatite, bone

## Abstract

Natural or synthetic hydroxyapatite (HA) has been frequently used as implant materials for orthopaedic and dental applications, showing excellent bioactivity, adequate mechanical rigidity and structure, osteoconductivity and angiogenic properties, no toxicity, and absence of inflammatory or antigenic reactions. HA can be easily synthesized or extracted from natural sources, such as bovine bone. However, the manufacturing costs to obtain HA are high, restricting the therapy. Herein, much effort has been paid for obtaning alternative natural sources for HA. The potential of HA extracted from skeleton of animals has been investigated. The aim of this review is to exploit the potential of HA derived from fish to fulfill biological activities for bone tissue engineering. In particular, HA from fish is easy to be manufactured regarding the majority of protocols that are based on the calcination method. Furthermore, the composition and structure of HA from fish were evaluated; the biomaterial showed good biocompatibility as a result of non-cytotoxicity and handling properties, demonstrating advantages in comparison with synthetic ones. Interestingly, another huge benefit brought by HA from bone fish is its positive effect for environment since this technique considerably reduces waste. Certainly, the process of transforming fish into HA is an environmentally friendly process and stands as a good chance for reducing costs of treatment in bone repair or replacement with little impact into the environment.

Bone lesions, either due to pathologies or traumas, are the most common large organ injury to humans (1). It is estimated that nine million of fractures occur annually worldwide, representing a major health problem in orthopedic clinics so far (2). Fortunately, bone tissue has the ability of healing by itself in most cases (1). However, in critical situations such as large traumatic bone defects, poor blood supply or in case of infections, the biological capacity of healing may be impaired, resulting in a delay of the consolidation process or even in non- union fractures (2).

In this context, the development of alternatives for effective bone reconstruction is one of the most clinically important long-term goals of research within the field of mineralized tissues. Gene therapy, tissue engineering and biomaterials have emerged as promising approaches for bone regeneration (3).

Biomaterials induce active biomineralization since they are able to induce bone growth (4). Therefore, clinical regenerative medicine has employed a number of materials, from natural to synthetic origins, in which the so-called bioactive materials are preferred due to their capacity to chemically adhere to bone tissue (4). Bioceramics represent a broad range of inorganic/non-metallic compositions including hydroxyapatite (HA), bioactive glass, calcium phosphate, and others (5). These materials have been extensively used in clinical practice, acting as fillers and scaffolds for bone reconstruction. Moreover, by promoting a tri-dimensional matrix that stabilizes and maintains the shape of the filled area, these biomaterials also support cell migration and angiogenesis, resulting in new bone formation during tissue repair (6).

During the last decades, many efforts have been devoted for obtaning alternative natural sources for biomaterials. Herein, the potential of HA extracted from skeleton of animals, including fishes, has been investigated (7). This is because million tons of fish are caught each year for human consumption worldwide (8). However, only 50 to 60% of the total catch is used; while the rest is discarded (9). It is important to stress that there is no commercial viable application for the use of the residues generated from fish markets around the world. Therefore, huge amounts of raw materials are wasted, culminating in undesirable environmental consequences. Moreover, fish waste is destined to landfill, which can cause serious problems to the human health (10). Thus, the conversion of waste obtained from fishing activity into biocompatible materials such as HA to be used for bone tissue engineering aplications, is an encouraging strategy that will minimize the environmental impact, and will incorporate more commercial value for this one. Therefore, the process of transforming waste bone fish into HA is an environmentally friendly process and stands as a good chance of reducing costs for treating bone repair with little impact into the environment (11).

The aim of the review was to present if HA derived from fish was able to fulfill biological activities for bone tissue engineering.

## Evidence acquisition

In this study, a comprehensive literature search for studies on HA and fish waste was performed. In brief, a search in PUBMED, MEDLINE, EMBASE, and Google scholar for all kind of articles (all publications to March 2018) was carried out in this setting. The following keywords were hydroxyapatite, bone, tissue engineering, fish, waste, human, animals, and/or experimental models. After searching the literature, only original papers were considered. Case reports and papers not written in English language were not considered in this study.

## Hydroxyapatite (HA)

HA, as its name suggest, is the hydroxylated representative of phosphate minerals known as apatites (HA chemical formula: Ca_10_(PO_4_)_6_(OH)_2_). These bioceramics crystalize into the hexagonal system, and can be artificially synthesized by different methods, including precipitation, hydrothermal, multiple emulsion, biomimetic deposition, and electrodeposition techniques (12). Also, HA powders and coatings have been successfully synthesized by the sol gel approach, in which a number of combinations between calcium and phosphorus precursors are mixed to produce a high pure HA at molecular levels (13).

Considering all the efforts that have been made to optimize its synthetic production, HA can also be extracted from bone matrix, where it is naturally found in abundance. Indeed, 60-70% of the acellular bone matrix consists of its inorganic components, which provide its significant mechanical strength. Natural HA exhibits a Ca/P ratio higher than 1.67 and its non-stoichiometric nanostructured crystal contains carbonate groups and traces of different ions such as HPO^2−^_4_, Na^+^, Mg^2+^, Sr^2+^, K^+^, Cl^−^ and F^−^ within its structure(14, 15).

Natural or synthetic HAs have been frequently used as a material for bone tissue engineering mainly due to their close composition similarity with natural bone (16, 17). It has been widely investigated as implant material for orthopaedic and dental applications, showing excellent bioactivity, adequate mechanical rigidity and structure, osteoconductivity and angiogenic properties, no toxicity, and absence of inflammatory or antigenic reactions (18). It is well known that, bone tissue binds directly to HA through a carbonated calcium deficient apatite layer at the bone-implant interface, inducing the deposition of newly formed bone after implantation (19). It has been demonstrated that HA surface supports osteoblastic cell adhesion, growth, and differentiation, and newly formed HA is deposited by the creeping substitution from the adjacent living bone (20). One of the advantages of HA is that its microstructure can be controlled to promote the formation of pores that allow the migration of blood vessels and bone tissue into the material (21).

Accumulating evidence indicates that HA plays an important role in bone metabolism, and may represent a possible substitute for autogenous bone grafts in the reconstruction of bone defects. For example, Jang et al. (22) assessed bone regeneration using two different types of HA: granular and porous, in extracted canine alveolar sockets. Osteogenic effect in both groups showed significantly higher bone-specific surface and bone mineral density in comparison with control group. Moreover, bone volume fraction, bone mineral density, and connective tissue amount related to disturbing osseointegration of the granular form was higher than the porous group. As a conclusion, the authors stated that HA is potentially a good bone substitute for alveolar socket healing. Silva et al. (21) evaluated the repair of calvarial bone defects in rats comparing autogenous bone grafts and porous bioceramic discs of HA/phosphate cement mixture. They found that the defects filled with bioceramic material were almost completely closed as a result of the joining of ceramic fragments and subsequent neoformed bone tissue following 24^th^ week after implantation. Orr et al. (23) found an increase in the modulus of elasticity in bone defects of rabbits implanted with a synthetic HA 26 weeks post-surgery. In oral and maxillofacial reconstruction, bovine HA represents one of the most important biomaterials for bone reconstruction for endosseous implants so far (24-26).

Considering the positive osteogenic effects of synthetic HA, some limitations merits attention, especially related to the high manufacturing costs (27). To overcome this problem, HA manufactured from natural sources rather than bovines may be a promising alternative for clinical applications. Natural materials are often more biocompatible since they offer a better interactive surface for cell attachment and growth (28). In fact, some authors stated that natural HA has better metabolic activity, and more dynamic response to the environment than the synthetic one (29).

## HA from fish: extraction and characterization

**Fig. 1:**
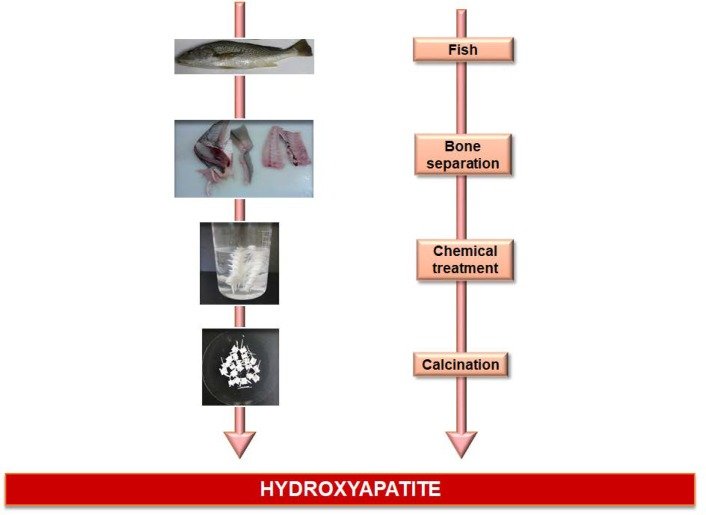
Chemical steps for extracting hydroxyapatite from fish

In the last years, HA from fish bone and scales has emerged as an alternative to substitute synthetic and bovine HA, because similar chemical properties have been achieved by simple and inexpensive methods (10, 11). It has been demonstrated that fish sources are safe and present low risks of disease transmission (10). Additionally, fishes are abundant in the environment, and the application of their byproducts is suitable for biomedical application since it would reduce environmental pollution and threats of biohazards to humans (11). Many different fish species have been used to obtain HA, such as salmon (10), carp, Japanese anchovy, sardine, tilefish, tuna (30), among others. For this purpose, many different protocols of extraction and methods for chemical analysis have been proposed in the literature, of which its goal is to obtain and to characterize HA extracted from fish (11). The majority of the protocols are based on the calcination method (Figure 1).

In 1995, Hamada et al. (30) have investigated the physico-chemical properties of HA extracted from 15 different species of fishes using the X-ray diffraction (XRD) and elemental analysis. It seems that, the calcination method is a simple, cheap, and effective method to extract HA. Bone samples were boiled in water, cleansed, dried at room temperature, and through an electric furnace, ashed at 600 ^o^C, for 3 h. After cooling, bone ashes were grounded in a mortar to a powder, and submitted to physico-chemical analysis. XRD analysis demonstrated characteristic peaks of HA in the samples from sea bream, horse mackerel, carp, shark, cattle, swine, and fowl, while the peak of tricalcium phosphate (TCP) was confirmed only in Japanese anchovy. Both peaks of HA and TCP were verified in sardine, mackerel, tilefish, croaker, triggerfish, lizard fish, Spanish mackerel, flying fish, congereel, and flat fish. Moreover, the analysis of elemental contents demonstrated that the most abundant element in the fish bones was calcium followed by phosphorus. Sodium and magnesium were present in some of the species as well.

Mustafa et al. (31) also used the calcination method to obtain HA from tilapia bones. Similarly, bones were boiled to remove adherent fish meat, dried and submitted to the crushing process to obtain powders with 0.2 mm size range. After that, the calcination process took place, and the raw powder was heated in furnace at temperatures ranging r from 800 °C to 1000 °C at a heating rate of 5 °C/min in 5 h. XRD analysis demonstrated the purity and stability of the powder. HA crystalline composition of tilapia bones for both raw powder and at 800°C were similar to the synthetic HA pattern.

In addition, Prabakaran and Rajeswari (32) utilized the calcination method to obtain HA from seer fish *(Thynnus thynnus)* using different temperatures (400 °C, 700 °C, 900 °C, and 1200 °C) for 2 h. As a result, authors observed that the temperature of 900^ o^C was responsible for producing the best HA phase. Besides, Venkatesan and Kim (33) evaluated different temperatures to calcinate fish bones (200oC to 1200 oC) for obtaining HA. Similar procedures were performed to clean bone samples, and 200 μm particle size of bone samples were placed in mold and heated in a furnace, at temperatures ranging from 200 °C to 1200°C. XRD analysis demonstrated that fish bone heated at 100 °C shows an amorphous nature, with low crystallinity of the HA. Under heat treatment at 400-900 °C, bone was initially transformed to a well-crystallized HA. The presence of TCP phases in the sample sintered at 1200 °C indicated the decomposition of HA. Thus, it is possible to develop pure phase HA at temperature below 1200 °C and a sintered composite of TCP/HA with heating at 1200°C.

Boutinguiza et al. (34) used the calcination technique to obtain HA from sword fish (*Xiphias gladius*) and tuna (*Thunnus thynnus*) bone. For this purpose, fish bones were boiled in water for 1 h and washed to remove the organic substances and adherent fish meat. Thereafter, bones were dried at room temperature for 24 h and calcined in a furnace at 600 and 950 °C. Once the calcination temperature had been reached, bones were maintained isothermally for 12 h, and then cooled at 20 °C/min and milled for use. The authors observed that HA presented a crystalline phase and a main composition of Ca and P, with an average Ca/P molar ratio around 1.87±0.02. Moreover, Na, Mg, potassium, and strontium were also detected in a much smaller amount, while the concentration of heavy metals in the obtained powders from fish bones was around 5 orders of magnitude lower.

Alkaline hydrolysis is another method commonly used to extract HA from tuna bone. It has been shown as a new strategy to obtain nanostructured HA (10). Venkatesan et al. (10) used both thermal calcination method and the alkaline hydrolysis method, and then they compared the products by means of chemical, physical, and microscopic analysis. For alkaline hydrolysis method, grounded tuna bone was treated with sodium hydroxide, at 250 ^o^C for 5 h for proper removal of organic moieties. The mixture was then filtered in a suction pump with continuous washing with water until the pH was neutral. The product was dried in an oven at 100 ^o^C. For thermal calcination method, tuna bone was placed in a silica crucible and subjected to a temperature at 900 ^o^C in an electrical muffle furnace in the presence of air atmosphere for 5 h. Charactherization was performed by thermal gravimetric analysis (TGA), Fourier transform infrared spectroscopy (FT-IR), XRD, microscopy, and transmission electron microscopy (TEM). FT-IR and TGA analysis showed that collagen and organic moieties have been eliminated by the proposed methods. XRD analysis demonstrated that thermally calcinated bone was more crystalline when compared to the alkaline hydrolyzed bone. TEM demonstrated that HA obtained from thermal calcination method was crystalline, with dimensions of 0.3-1.0 µm, whereas HA obtained through the alkaline hydrolysis method was structured in nanocrystals, with 17-71 nm length and 5-10 nm width, respectively. As a conclusion, authors stated that alkaline hydrolysis is the best approach for nanostructured arrangement rather than the thermal calcination method. Overall, alkaline hydrolysis method takes an advantage of deriving HA with required physicochemical properties. This could support several biomedical applications. Fish heads from *Argyrosomus regius* were conducted to define chemical composition, morphology, and crystallography. The results showed that the powder was mainly composed of aragonite (CaCO3) and calcite (CaCO3) (35).

In an earlier study, Venkatesan et al*.* (36) obtained HA from salmon fish using the alkaline hydrolysis method again. Salmon fish bones were boiled at 200 °C for 3 h in water, and afterwards in acetone and 2% NaOH for 1 h to remove soft tissues. Bones were crushed with a mortar and pestle. For HA isolation, crushed bones were heated with NaOH at 200^o^C to remove the organic part. HA was collected into conical tubes, centrifuged and washed with H_2_O until it reached neutral pH, and was dried in an oven at 100 ^o^C. XRD analysis demonstrated that nano-HA from salmon fish was amorphous. Microscopic analysis demonstrated that isolated nano-HA possessed a nano- structure with a size range of 6–37 nm.

Fish scales have also been used as a source for HA extraction (37, 38). Mondal et al. (39) collected fish scales from fresh water fish (*Labeo rohita*), and submitted them to a process to deproteinize the material through external washing with HCl for 24 h. Thus, fish scales were washed several times, and treated with NaOH solution. The remaining material was dried at 60 °C and calcined to different temperatures for producing HA ceramics. XRD patterns of synthesized HA demonstrated sharp peaks of powders at high calcination temperature confirming the complete crystallization of the powder.

Piccirillo et al. (40) studied apatite- and tricalcium phosphate-based materials from codfish bones. Bones were annealed at temperatures between 900 and 1200 °C, giving a biphasic material of HA and TCP. The treatment of bones in solution prior to annealing changed the composition of the biomaterial. Single phase HA, chlorapatite (Ca10(PO4)6Cl2), and fluorapatite (Ca10(PO4)6F2) were obtained using CaCl_2_ and NaF solutions, respectively.

Pon-On et al. (38) have investigated the physicochemical, bioactivity and biological properties of HA derived from fish scale as far as to compare with synthesized HA obtained by co-precipitation from chemical solution as a standard. The analysis showed that the HA from fish scale was composed of flat-plate nanocrystal with a narrow width size of about 15-20 nm and having a range of 100 nm in length, and that the calcium phosphate ratio (Ca/P) was 2.01 (Ca-rich CaP), while synthesized HA consisted of sub-micron HA particle having a Ca/P ratio of 1.65. Bioactivity test showed that the HA formed more new apatite than synthetic HA after being incubated in simulated body fluid for 7 days.

Recently, our research group has synthesized HA from whitemouth croaker fish (39). After washing and alkali treatment, fish bones were exposed to calcination at 800 °C. XRD analysis, infrared spectroscopy, and SEM revealed the presence of HA (39).

## HA from fish: biologial activities for bone tissue engineering

Some studies have described the potential benefits of HA extracted from fish for bone tissue engineering applications. The results are summarized in Table 1. *In vitro* tests revealed no signs of cytotoxicity and increased mineralization in the presence of HA derived from bone and fish scales (10, 11). Bioactivity tests showed that fish HA forms more new apatite than does the synthetic HA after being incubated in simulated body fluid (SBF) for 7 days (36,38). Moreover, some authors demonstrated a positive effect of HA on the viability and alkaline phosphatase activity in osteoblast cells (38, 40, 42, 43).

*In vivo* test using murine models, demonstrated that HA from fish waste has good biocompatibility as a result of absence of cyto and genotoxicity (39). Venkatesan and Kim (33) also demonstrated no cytotoxicity of HA in MG63 cells *in vitro*. Similarly, Boutinguiza et al. (34) investigated the effects of HA obtained from sword fish and tuna bones, which are available as waste from fishing activities. *In vitro* cytotoxicity test revealed that all materials were non-cytotoxic.

**Table 1:** *In vitro* and *in vivo* studies of HA extract from fish for bone tissue engineering applications in chronological order

**Biomaterial source** **(HA and composites)**	**Experimental model**	**Analysis**	**Results**	**References**
HA crystals isolated from the saltwater fish, tuna (*Thunnus obesus*), bone	Human osteosarcoma cell line (MG-63) treated with different concentrations of micro and nanocrystals of HA at the cell culture medium.	Morphology (microscopy)Cytotoxicity, and cell proliferation (MTT)	Limited and lesser cell growth on treated HA groups. Slower cell proliferation rate on HA treated (versus control). Micro and nanoparticles had similar cytotoxicity.	
HA from sword fish (*Xiphias gladius*) and tuna (*Thunnus thynnus*)	Mouse calvaria MC3T3-E1 cells.	SEM, FTIR, TEM. Cell viability	HA with crystalline phase. Higher cell viability	
HA from fish (*Labeo rohita*) scale	RAW macrophage- like cell line seeded on HA surfaces. HA powder samples of different weights (100, 200, 400 lg) were placed into culture media. Bone defects in Wistar rats	*In vitro*: cytotoxicity evaluation and MTT assay, SEM images of attached cells on to the HA surface. *In vivo*: histological analysis	*In vitro*: no cytotoxic effects, cellular attachment and proliferation on HA surface. *In vivo*: bone formation and cell infiltration and integration in HA fillers.	
Decellularized fish scales (type I collagen and HA)	Myoblastic cell line (C2C12) cultured on the acellular fish scalesA bone pin made of decellularized fish scales used for the internal fixation of femur fractures in New Zealand rabbits.	*In vitro*: growth curve (biocompatibility), cell morphology (SEM). *In vivo*: Periodic X-ray evaluations and histologic examinations postoperatively.	*In vitro*: great adhesion and good cell growth. *In vivo*: improvement of the fracture healing, integration with the adjacent tissue and material degradation with time.	
HA from fish (Carassius auratus) scales	MC3T3-E1 osteoblastic cell culture		High cytocompatibility, and the ability to guide cell proliferation and migration along the scale ridge channels of the fish scales.	
HA from fresh water fish (*Labeo rohita *and* Catla catla*) scales HA synthesized from SBF solution	MSCs seeded over HA scaffolds.	Cell viability (MTT assay), proliferation study (DNA quantification), and microscopy image	Attachment, growth and proliferation of MSCs over the prepared HA scaffolds.	
HA from salmon	MSCs	FT-IR, XRD, and SEM. *In vitro*: toxicity and mineralization	Presence of a carbonated group, similar to synthetic HA, with an amorphous feature, sized 6–37 nm. Non-cytotoxicic and higher mineralization	
HA and biphasic material HA/β- TCP from Atlantic cod fish bones	Culture with osteosarcoma Saos-2 cell line	Cytotoxicity, bioactivity, and haemocompatibility assays.	Materials were not cytotoxic, non-haemolytic. They supported cell growth and crystal formation.	
HA from fish scale and synthetic HA	Material incubation in SBF and culture with osteoblast like cells.	Bioactivity and biocompatibility assays. MTT and ALP analysis	More new apatite formed in fish HA after incubation. Higher cell adhesion on the HA fish surface. Cells were able to spread. Significant increases in the proliferation and activity of osteoblasts over fish HA scaffolds. Biomaterials derived from fish scale are biologically better than the chemically synthesized HA.	
Three types of collagen scaffolds(collagen, collagen-chitosan, and collagen-HA	Blue shark	physico-functional and mechanical properties in relation to biocompatibility and osteogenesis	Higher level of ALP induced by collagen-hydroxyapatite scaffold. Addition of chitosan and HA improved the stiffness and degradation rate, but lowered the water binding capacity and porosity of the scaffold.	
HA from whitemouth croaker fish	*S*ubcutaneous test	Histopathology Cytotoxicity Genotoxicity in multiple organs	Good biocompatibility Absence of cyto-and genotoxicity	

ALP: alkaline phosphatase; FT-IR: Fourier transform infrared spectroscopy; HA: hydroxyl apatite; MSCs: mesenchymal stem cells; SEM: scanning electron microscopy; SBF: synthetic body fluid; TEM: transmission electron microscopy; TCP: tricalcium phosphate; XRD: X-ray diffraction.

Recently, Piccirilo et al. (44) have studied the biological properties, including cytotoxicity, bioactivity and haemocompatibility of single phase HA and biphasic material HAe/β-tricalcium phosphate (HAp/β-TCP) obtained from Atlantic cod fish bones. *In vitro* results using Saos-2 cells showed that these materials are not cytotoxic, neither in their powder nor in pellet form. Moreover, crystal formation was observed on the surface of both materials when immersed in fetal bovine serum. Overall, these results confirm the suitability of these materials for biomedical applications.

Venkatesan et al. (36) also observed that HA from salmon interacted positively with mesenchymal stem cells (MSCs), presenting non-toxicity. Increased mineralization was observed for nano-HA treated MSCs compared with the control group, suggesting that HA nanoparticles derived from salmon are promising biomaterials in the field of bone tissue engineering.

In addition, collagen HA was prepared from the cartilage of blue shark and investigated for their physico-functional and mechanical properties in relation to biocompatibility and osteogenesis. HA improved the stiffness and degradation rate, but lowered the water binding capacity, and porosity of the scaffold. Osteogenesis study revealed that the addition of HA to collagen scaffolds could efficiently promote osteoblast cell formation (45).

Fang et al. (42) studied the structure and the cytocompatibility of fish scales from *Carassius auratus*, and observed that the main components of scale were HA and type I collagen fibers. Moreover, *in vitro* results demonstrated that HA from fish scales were biocompatible and able of increasing MC3T3 cell proliferation. Panda et al. (46) extracted and characterized HA from fish scale, and showed that synthesized HA consists of sub-micron HA particles. MTT assay and quantitative DNA analysis showed growth and proliferation of cells over the HA scaffold with the increase in time. The shape and size (morphology) of MSCs after 3 days showed a transition from rounded shape to elongated and flattened shape, expressing its spreading behavior. These results confirm that HA biomaterials from fish scale are physico-chemically and biologically equivalent to the chemically synthesized HA from SBF. Biological HA, thus, possesses a great potential for conversion of industrial byproduct into highly valuable compounds using simple effective and novel processes.

**Fig. 2:**
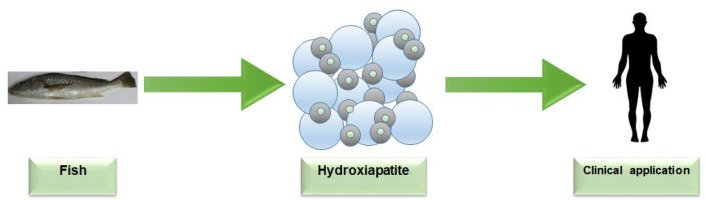
Fish, hydroxyapatite and clinical application

## Future Perspectives

This paper presents recent data regarding the use of HA from fish for tissue engineering, focusing on studies concerning its physical-chemical properties and biological response. These findings suggest that HA from fish plays an important role with respect to inducing osteogenic activities, supporting cell growth *in vitro*, with a low risk of transmission of infection-causing agents as far as good biocompatibility (42, 43).

In this context, HA from fish is a promising resource for bone tissue engineering with commercial interest, and for medical and dental products (Figure 2). However, some limitations and challenges for their use should be overcome. Among them, all biocompatibility tests should be conducted through in vitro and in vivo studies using different species of fishes in order to determine the safety and efficiency of the material.

Furthermore, different forms and presentation of HA from fish should be manufactured and tested. Recent studies have demonstrated that nano-sized HA can mimic better dimensions the components of bone tissue (47). Thus, nano-HA fish-based biomaterials may present the same advantages of nano synthetic HA, offering a better bioactivity and dissolution than coarser crystals due to large surface to volume ratio and unique chemical properties (48). Therefore, it is expected that nano-HA from fish would promote increased osteoblast adhesion and cell proliferation (49).

## Conclusion

In this review, we present all published results reporting the use of HA from fish for tissue engineering. As stated above, HA derived from bone fish is easy to be manufactured, and present good biocompatibility as a result of non-citotoxicity and handling properties, demonstrating advantages in comparison to synthetic HA. Another huge benefit brought by bone fish HA is its positive effect for environment, because the procedure potentially reduces waste. However, further studies are welcomed in light of biocompatibility and biological effects especially using *in vivo* models before this biomaterial can be used with safety in the clinical setting (Figure 2). Therefore, this is an area that warrants further investigation to ensure if HA from fish can be better employed, resulting not only in the aggregation of commercial value to the waste produced by humans, but also as a great benefit, which is a cleaner environment in the future.

## Conflict of Interest

None declared.
